# Research Progress of Oral Immune Tolerance Mechanism Induced by Whey Protein

**DOI:** 10.3390/nu17091517

**Published:** 2025-04-29

**Authors:** Mao Lin, Qianqian Zhang, Yanjun Cong

**Affiliations:** College of Food and Health, Beijing Technology and Business University, Beijing 100048, China

**Keywords:** cow milk allergy, oral immune tolerance mechanism, hydrolyzed whey protein, hypoallergenicity

## Abstract

Cow milk allergy (CMA) is prevalently observed among infants and young children, exerting adverse effects on their growth and quality of life. Oral immune tolerance (OIT) is a more effective method for the prevention and treatment of CMA. The site of OIT is mainly in the gastrointestinal tract, so this article reviews the composition and structural characteristics of intestinal immune system, the molecular mechanisms of immune tolerance by regulatory T cells (Treg), dendritic cells, and gut microbiota. In addition, this paper summarizes the research progress of T cell epitope peptides of β-lactoglobulin and α-lactalbumin in whey protein hydrolysates. The mechanism of OIT induced by whey protein hydrolysate or whey protein combined with other anti-allergic components (phenolic compounds, probiotics, etc.) is overviewed to provide new ideas for the development of hypoallergenic infant formula.

## 1. Introduction

Food allergy (FA) diseases usually occur in infancy and early childhood [1]. IgE-mediated FA affects 8% of children and 10% of adults in the United States [2]. CMA is one of the most common food allergies according to the World Allergy Organization (WAO) Diagnostic and Action Criteria for CMA (DRACMA) guidelines in 2022. The prevalence in infants is about 2–4.5% [3]. CMA is an immune-mediated adverse reaction to one or more milk proteins, which is mainly manifested as adverse reactions to α-lactalbumin (α-LA) and β-lactoglobulin (β-LG) in whey proteins [4]. In China, researchers from Children’s Hospital of Chongqing Medical University conducted a cross-sectional survey on the trend of FA in the region from 1999 to 2019 and found that the prevalence of CMA increased from 1.6% to 5.7% [5]. Therefore, the prevention and treatment of CMA is imperative.

At present, methods for the prevention and treatment of FA mainly include avoidance therapy, food processing, and specific immunotherapy [6]. As part of the routine management of FA, avoidance therapy is based on the prevention of allergic reactions by avoiding food intake and accidental exposure to allergens. However, this treatment is not suitable for patients and their families [7,8], and the diet of eliminating foods containing allergenic proteins (milk, eggs, peanuts, and nuts) may lead to nutritional imbalance, cause anxiety, and reduce the quality of life [9]. Food processing is the use of certain processing methods (such as enzymatic hydrolysis, heat treatment, and ultrafiltration) to change the structure or lose the activity of milk protein, which can reduce but not eliminate its sensitization [10,11]. According to the route of administration, specific immunotherapy is divided into subcutaneous immunotherapy, oral immunotherapy, and sublingual immunotherapy, among which oral immunotherapy has been widely studied in FA [12]. OIT specifically inhibits cellular and humoral immune responses by reapplying antigens via the oral route and prevents specific immune responses in the gastrointestinal tract from proteins exposed orally by individuals or animals [13]. OIT is an established and successfully used FA treatment program [14]. Oral immunotherapy using allergen proteins has made some progress in controlling major food allergies such as peanuts, egg and milk [15,16,17,18]. However, adverse events caused by the excessive intake of allergenic proteins have also been reported [19]. In two high-dose oral cow’s milk immunotherapies, 15.8–43% of patients showed allergic reactions during OIT [20,21]. Because the allergen protein contains both T and B cell epitopes, whereas B cell epitope peptides can cause allergic [22,23]. To avoid the development of underlying allergic symptoms, it is important to study hydrolyzed peptides of allergen proteins containing T-cell epitopes, which may trigger the development of immune tolerance (IT) when received by antigen-presenting cells (APCs) in the gut [24].

The goal of FA prevention and treatment is to induce continuous desensitization to food allergens or long-term OIT [25]. Therefore, the development of therapeutic products to prevent allergic reactions, especially for the most severe ones, is of great significance [9,26].

## 2. Mechanisms of Oral Immune Tolerance

After repeated exposure to or consumption of small amounts of food allergens, the body will establish IT [27]. IT means that the human body does not show strong allergy after eating food allergens but shows local or systemic hyposensitivity or no reaction [28]. Failure of the body to establish IT will lead to FA and even develop diseases such as allergic asthma and enteritis [29,30]. The reported mechanism of OIT involves the gastrointestinal barrier, various immune cells, and humoral factors [31].

### 2.1. Composition and Structural Characteristics of Intestinal Immune System

The gastrointestinal tract is exposed to many food allergens, which protects the human body through the intestinal mucosal immune system and enables the host to achieve IT. The proximal mucosa of the intestinal lumen consists of a single layer of columnar epithelial cells and the underlying lamina propria (LP) and contains the vast majority of immune cells [32]. Gut-associated lymphoid tissue (GALT) is the main site for the initiation and differentiation of adaptive immune cells, and mesenteric lymph nodes (MLNs) and Peyer’s patch (PP) are the main components of GALT [33]. Intestinal epithelial cells, dendric cells (DCs), microwrinkled cells, and macrophages are diffused here, which are responsible for antigen uptake, processing, and presentation [34]. In addition, regulatory T cells (Treg) play an important role in maintaining antigen tolerance by inhibiting the immune response [35,36,37].

### 2.2. Regulatory T Cells and Oral Immune Tolerance

Treg are a special subset of CD4+ cells, which play a crucial role in the establishment and maintenance of immune homeostasis [38]. The oral induction of Treg occurs in the small intestine [39]. Repeated low doses of allergen are beneficial to induce IT in Treg, which is the main mechanism of OIT. However, the loss and anergy of T cells mainly occur during the establishment of OIT to high doses of food allergens [40]. Treg can be divided into two major categories: one is the natural regulatory T cells (nTreg). The second is induced regulatory T cells (iTreg) [41]. nTregs are mainly CD4+CD25+T cells with a high expression of Forkhead box P3 (Foxp3), which can participate in the formation of IT by inhibiting the transcription factors of T helper 1 cell (Th1) and T helper 2 cell (Th2) polarization. iTreg develops from peripheral naive T cells and can express Foxp3+, transforming growth factor-β (TGF-β), and interleukin-10 (IL-10) [42]. In MLN, DCs secrete indoleamine 2,3-dioxygenase, IL-10, and retinoic acid (RA) [43]. All three classes of factors can differentiate naive T cells into Treg. Treg enters the intestinal LP and inhibits the activation of effector cells related to FA inflammation, such as Th2 and their related factors, mast cells and their secreted factors, and eosinophils [44].

#### 2.2.1. Foxp3+CD4+T Cells

Foxp3 is specifically expressed in Treg. It is a transcription factor necessary for the induction of Treg development and has the function of maintaining the proliferation and differentiation of Treg. Because of its unique role, Foxp3 is considered as a hallmark molecule of Treg [45]. Although most Foxp3+ Tregs were initially thought to originate in the thymus, it was later shown that native CD4+ T cells can become Foxp3+ Treg cells in peripheral tissues [46]. Foxp3+CD4+T cells from the thymus can regulate the activity of other T cells, and it can play an important role in maintaining peripheral tolerance by suppressing the response of mature T cells to allergens [47,48]. Increased Foxp3 mRNA expression during cow’s milk protein-specific induction of oral immune tolerance (CMP-OIT) predicts faster acquisition of tolerance in CMA infants [49]. Overall, effective CMP-OIT successfully promoted expansion of casein-specific, functionally stable Foxp3(+) Treg cells while reducing Th2 responses in children developing OIT [50]. OIT may be achieved by blocking IgE-mediated allergen presentation and significantly increasing IL-10 cytokine responses, TGF-β cytokine responses, and Treg functions [51].

#### 2.2.2. Th3 Regulates Cells

A different type of regulatory CD4+T cell is known as Th3 cells [52]. They have also been found to play an important role in OIT [53]. Th3 regulatory T cells are dependent on TGF-β and express latency-associated peptide on their surface [54]. Their secreted Foxp3 can induce Foxp3+CD4+T cell production [55]. Studies have shown that the expressions of latency-associated peptide and IL-10 are potential biomarkers of oral tolerance during induction of OIT by plant antigens [56,57].

#### 2.2.3. Tr1 Cells

Tr1 cells are another regulatory CD4+T lymphocyte subtype induced in the periphery. The tolerant properties of Tr1 cells are related to the release of IL-10 and TGF-β upon T cell antigen receptor activation by antigens [58]. Tr1 cell induction or cell therapy can be used to prevent autoimmune diseases or transplant rejection [59]. TGF-βinduced differentiation of Tr1 cells was observed in a gliadin tolerance mouse model [60]. The results of Bergerson et al. showed that the proportion of Tr1 cells in normal children was higher than that in children with FA [61]. This suggests that Tr1 cells are important in OIT, especially in young children.

### 2.3. Relationship Between Dendritic Cells (DCs) and Oral Immune Tolerance

**Figure 1:**
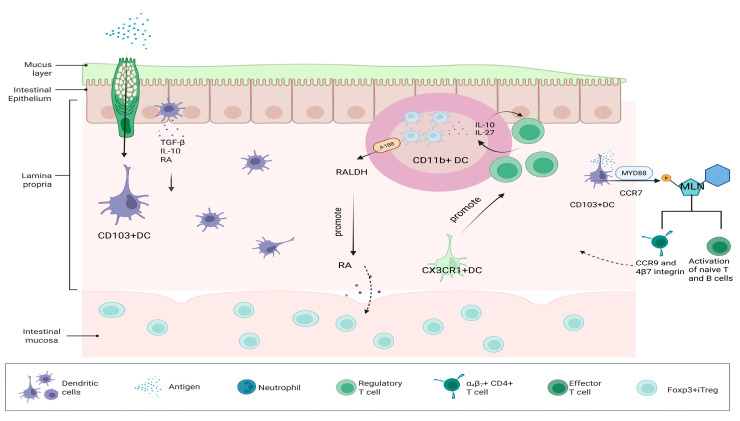
Diagram of the mechanisms by which three major DC subsets induce OIT.

DCs are professional APCs, which are essential for T cell activation and play an important role in the induction and maintenance of OIT. In the intestinal mucosa, DCs are diffusely distributed in the LP and GALT (mainly including PP and MLNs) [62,63]. There are three major DC subsets in the small intestine, which are CD103+DCs, CD11b+DCs, and CX3CR1+DCs (Figure 1) [64]. At steady state, small intestinal goblet cells act as channels to deliver low molecular weight (MW) soluble antigens from the intestinal lumen to CD103+ DC in the underlying LP. The preferential delivery of antigens to tolerogenic DC suggests that DC plays a key role in intestinal immune homeostasis [65]. A major DC subset in the intestinal LP is CD103+ DC, which constitutively migrates to the MLN, where it promotes tolerogenic responses [66,67]. MyD88 signaling is required for optimal CCR7-dependent steady-state migration of LP CD103+DC to the MLN [68]. CD103+DCs can produce RA, IL-10, TGF-β, and other related factors in the gut, and these three factors are key to the development of tolerance, so they are considered to be key determinants of the tolerance environment of the intestinal mucosa [69,70]. Intestinal CD103+DCs are the only DCs in the intestine that can present food proteins and bacterial antigens to T cells [71]. CD103+DC dendritic cells can also take up antigens and migrate to MLN, where they can stimulate the activation of naive T cells and B cells [72]. They can also express homing factors such as CCR9 and 4β7 integrin and migrate to the LP of the intestine [73]. Due to the dominant expression of B7-H1 and B7-DC, DC in MLN enhanced the specific-antigen generation of CD4+Foxp3+ inducible regulatory T cells (iTregs) but not CD4+ effector T cells in CD4+Foxp3 T cells [74]. This also has important implications for OIT. CD11b+ DCs in PP are a major source of IL-10 and IL-27, increased by interaction with antigen-specific T cells in PP, and these DCs act as inducers of IL-10 producing T cells in oral tolerance [75]. The co-signaling molecule 4-1BB with CD103+CD11b+ DC promotes OIT through high expression of retinal dehydrogenase (RALDH), an enzyme that promotes RA and contributes to the differentiation of Foxp3+ iTreg in the intestinal mucosa [76,77]. p38α signaling is a central pathway for programming the tolerogenic activity of mucosal CD103+ DCs [78]. Compared with CD103+DCs, CX3CR1+DCs do not express chemokine CCR7 and, therefore, cannot migrate to MLN but can promote the local specific proliferation of Treg cells in the LP [79]. Dawicki W et al.’s animal experimental data showed that regulatory DC immunotherapy was effective in FA and suggested that the induction of Foxp3+ Treg may be a useful strategy for inducing OIT [80].

### 2.4. Relationship Between Gut Microbiota and Oral Immune Tolerance

**Figure 2:**
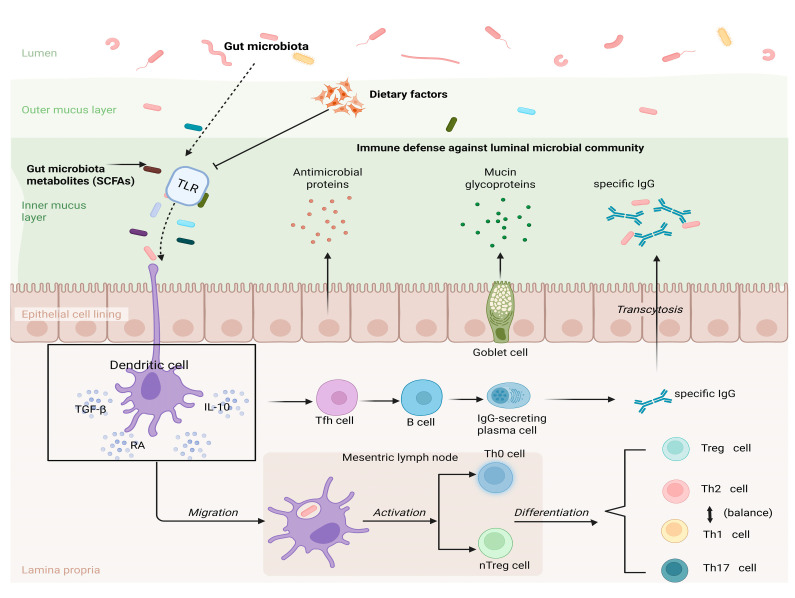
Microbiota–DC–Treg axis.

Gut microbiota refers to the various microorganisms that live in the human gut. Intestinal microorganisms participate in immune regulatory responses through DC and activate DC present on the surface of the intestinal mucosa through the Toll-like receptor (TLR) pathway, thereby promoting the differentiation of Treg [81]. These activated cells produce cytokines that, in turn, activate nTreg or Th0 cells, allowing them to mature into the corresponding T cell subtypes, Th1, Th2, Th17, or Treg (Figure 2). In healthy individuals, all Th cell subsets are in homeostasis with Treg [82]. In patients with FA, however, this balance is disrupted by antigens, and tolerance needs to be re-established. Gut microbiota may affect FA susceptibility through a variety of mechanisms. Mouse models of FA have shown several roles of gut microbiota, including the modulation of Th2 immune responses, developmental regulation of mucosal immunity and oral tolerance, modulation of basophil populations, and promotion of gut barrier function by reducing intestinal permeability and increasing mucus production [83]. In the process of establishing OIT, the regulation mechanism of intestinal flora is mainly achieved by increasing protective Treg and enhancing intestinal mucosal barrier [84]. The critical time to build IT is early in life [85,86].

Related studies on probiotics in regulating intestinal flora to induce OIT have also been reported. Probiotics are living microorganisms that provide benefits to host health by colonizing the body when given in sufficient amounts [87]. Three probiotic strains, Lactobacillus rhamnosus LA305, Lactobacillus sialica LA307, and Bifidobacterium longum subsp LA308, exert different preventive effects in a CMA mouse model [88]. These three probiotic strains affect gut bacterial communities and alter immune and inflammatory responses, leading to tolerance. Moreover, all three strains have direct effects on DC, which play an important role in food sensitization through their potential tolerance and anergy. Prebiotics, as the “food” of probiotics, also play a certain promoting role in the establishment of OIT. The experimental data of Rumiko Shibata et al. [89] demonstrate that 1-ketoseinduces IT to milk protein in children with CMA by enhancing the abundance of Clostridium Fusicatenibacter spp. in the intestinal flora. It has been reported that this bacterium can induce immune cells to generate IL-10 and was inversely correlated with antigen-specific IgE levels in serum [90]. Consequently, probiotics play a significant role in inhibiting the production of IgE and inducing IT to allergens in CMA.

## 3. T Cell Epitopes of Whey Protein Induce Immune Tolerance

Whey protein is a by-product of cow milk to produce cheese. It is a very important functional raw material and is widely used in the food and medical industries, including infant formula (IFs), baked goods, sports nutrition, and medical foods. It is mainly composed of β-LG (50–55%), α-LA (20–25%), glycomacropeptide (10–15%), bovine serum albumin (5–10%), immunoglobulin (10–15%), lactoferrin (1–2%), and a few microcomponents [91], among which β-LG and α-LA are considered as the major allergens [92].

To manage or prevent CMA, hypoallergenic whey protein materials need to be developed. Its core principle is to hydrolyze linear epitopes of whey protein through protease [93]. The exploration of allergen hydrolysates enriched with T cell epitopes with OIT will become a hot spot and a difficult point in research. It has been found that infant formula based on hydrolyzed whey protein shows higher peripheral blood mononuclear cell (PBMC) and cord blood mononuclear cell (CBMC) proliferation capacity compared with IFs based on casein, because of the presence of T cell epitopes and stability in the whey hydrolysate [94,95].

T cell epitopes of allergens are sorts of short peptides composed of 12–26 consecutive amino acids that activate naive T cells by MHC class II molecules expressed on APCs [96,97]. These fragments lack secondary or tertiary structure and do not cross-link IgE or activate effector cells. They have regulatory potential in reshaping T cells from a Th2 type to a Th1 and/or Treg-dominated response. These epitopes may trigger the development of tolerance when accepted by APCs in the gut [24]. These properties give the T cell epitope potential as an immunomodulatory peptide for OIT to CMA.

At present, more effective methods to identify T cell epitopes mainly include a T cell proliferation test and animal experiment. Bioinformatics has been applied to the prediction of T cell epitopes because of its convenience and speed for a long time, but the prediction results need to be further verified by experimental data [98]. T-cell proliferation assay exploits the principle that CD4+T cells proliferate on a large scale in response to antigen either in vitro or in vivo. Lewis and Sloan et al. [99] verified that there were some differences in the phenotype of CM+ T cells in CMA patients and determined that the increase in CM+ Foxp3+ cells was a potential diagnostic biomarker. There is a Th1/Th2 balance in normal human body. When the body has an allergic reaction, this balance is broken. Therefore, T cell epitopes can be identified by changes in factors related to T cell proliferation.

There are many studies on the immune tolerance effect of β-LG hydrolysate (Table 1). Totsuka et al. [100] synthesized a set of overlapping peptides of β-LG with a length of 15 amino acids, a total of 148 peptides, and then performed mouse lymphocyte proliferation experiments to determine three different strains of mouse T cell epitopes. Three major T cell epitopes (AA67–75, AA71–79, and AA80–88) were identified in BALB/c mice. C57BL/6 mice were used to identify a major T cell epitope AA115–136. Two major T cell epitopes, AA131–154 and AA86–105, were mapped using C3H/He mice. Inoue R et al. [101] used the whole β-LG sequence as a template, synthesized peptides with 12–21 amino acids in length according to Fmoc solid-phase synthesis method, then established β-LG specific T cell lines (TCLs) from PBMC of allergic patients, and detected the proliferation of T cells induced by antigen. Thus, three major T cell epitopes (AA1–21, AA47–67, and AA97–117) were identified. The experimental results of Sakaguchi et al. [102] showed that BLG101-BLG112 (KYLFCMENSAE) is likely to be one of the core sequences of β-LG T cell epitopes. Kondo et al. [103] investigated the response of bovine β-LG specific T cell clones to the single amino acid substitution of the T cell core epitope AA97–117 and identified BLG102-BLG112 as the minimal essential region for BLG97-BLG117. Amino acids E108 and C106 were found to be essential for T cell responses. This result was also verified in a subsequent experiment by Hiroshi M. Ueno et al. [104]. Joost W. Gouw et al. [105] identified HLA-DRB1-restricted peptides associated with AA11–0 and AA23–39 of β-LG, and synthesized peptides were recognized by cow’s milk protein-specific TCLs and induced T cell proliferation. Zihao Xu et al. [106] evaluated AA1–20, AA24–50, and AA123–139 of β-LG as potential hypoallergenic peptides by cell model, which could be used as candidate peptides for inducing IT of CMA.

An animal model of FA was established, and then measure serum levels of specific antibodies, cytokines (IL-10, TGF-β), chitinase 3-like protein-1 (CHI3L1), and histamine secretion or cell proliferation production to identify T cell epitopes [107]. Koko Mizumachi et al. [108] investigated whether β-LG peptides (AA42–56, AA62–76, and AA139–154) could be orally tolerated. The results showed that all three peptides successfully inhibited the proliferation of T cells. A unique tolerogenic peptide AA139–154 was identified in the mouse model, which could inhibit T and B cell responses to β-LG. Thang et al. [109] sensitized mice with two dominant β-LG epitopes (AA67–88 and AA139–153) by gavage of β-LG and found that the symptoms of allergic reaction in mice were relieved. The experimental results of Kostadinova et al. [110] showed that β-LG-derived peptides AA29–45, AA35–52, AA41–58, and AA47–64 may have OIT. However, not all T cell epitopes can improve IT. Meulenbroek et al. [111] found that T cell epitopes (AA47–64) of β-LG can reduce acute allergic skin reactions. The percentage of CD11b+CD103+ dendritic cells and CD25+Foxp3+T cells were also increased to enhance OIT, but the T cell epitopes AA92–100 and AA91–108 did not show IT. Tian linghan et al. [112] screened β-LG hydrolysate with OIT through vitro and in vivo experiments and identified the polypeptide sequences with potential T cell IT, which were AA13–29, AA26–46, AA55–69, AA83–108, AA110–128, and AA154–174, respectively. The results indicate that the hydrolysates containing T cell epitopes are not necessarily OIT, which needs to be further verified by clinical experiments.

Few studies on the immune tolerance of α-LA have been reported. Meulenbroek et al. [113] synthesized 19 peptides using the amino acid sequences of α-LA as a template using the solid-phase method. The specificity of peptides for α-LA was verified by using milk specific TCLs. Four peptides (AA 19–36, AA25–42, AA31–48 and AA43–60) induced the proliferation of two cow’s milk-specific TCLs. Zihao Xu et al. [106] evaluated three peptides (AA29–51, AA80–90, and AA94–103) of α-LA as potential hypoallergenic peptides by cell model, which could be used as candidate peptides for inducing IT of CMA. Xumei Wang et al. [114] identified potential allergenic peptides and key amino acids in the digested products of glycosylated α-whey protein through sensitization analysis and molecular docking. The results showed that AA94–104 were tightly bound to MHC molecules, but whether it was immune tolerant or not, the results of further experiments were not reported.

**Table 1:** Summary of T cell epitopes of β-LG and α-LA in whey protein.

Allergen Name	T Cell Epitopes	Research Methods	References
Beta-lactoglobulin	AQKKIIAEK(67–75), IIAEKTKIP(71–79), AVFKIDALN(80–88), QSLVCQCLVRTPEVDDEALEKF(115–136), EALEKFDKALKALPMHIRLSFNPT(131–154), ALNENKVLVLDTDYKKYLLF986-105)	Mouse lymphocyte proliferation experiments	[100]
	LIVTQTMKGLDIQKVAGTWYS(1–21), KPTPEGDLEILLQKWENDECA(47–67), TDYKKYLLFCMENSAEPEQSL(97–117)	Allergic patients T cell proliferation test	[101]
	KYLFCMENSAE(101–112)	Allergic patients T cell proliferation test	[102,103,104]
	DIQKVAGTWYSLAMAASDIS(11–30), AMAASDISLLDAQSAPL(23–39)	Allergic patients T cell proliferation test	[105]
	LIVTQTMKGLDIQKVAGTWY(1–20), MAASDISLLDAQSAPLRVYVEELKPTP(24–50), VRTPEVDDEALEKFDKA(123–139)	KU812 cells cultured with patient serum	[106]
	YVEELKPTPEGDLEI(42–56), ENDEAAQKKIIAEKT(62–76), ALKALPMHIRLSFNPT(139–154)	Mouse animal experiment	[108]
	AQKKIIAEKTKIPAVFKIDALN(67–88), ALKALPMHIRLSFNP(139–153)	Mouse animal experiment	[109]
	QKVAGTWYSLAMAASDIS(29–45), WYSLAMAASDISLLDAQS(35–52), WYSLAMAASDISLLDAQS(41–58), LLDAQSAPLRVYVEELKP(47–64)	Mouse animal experiment	[110,111]
	GAQALIVTQTMKGLDIQ(13–29), LDIQKVAGTWYSLAMAASDIS(26–46), LRVYVEELKPTPEGD(55–69), AQKKIIAEKTKIPAVFKIDALNENKV(83–108), VLDTDYKKYLLFCMENSAE(110–128), KALKALPMHIRLSFNPTQLE(154–174)	Mouse animal experiment	[112]
Alpha-lactalbumin	GGVSLPEWVCTTFHTSGY(19–36), EWVCTTFHTSGYDTQAIV(25–42), FHTSGYDTQAIVQNNDST(31–48), QNNDSTEYGLFQINNKIW(43–60)	Allergic patients T cell proliferation test	[113]
	TTFHTSGYDTQAIVQNNDSTEYG(29–51), FLDDDLTDDIM(80–90), KILDKVGINY(94–103)	KU812 cells cultured with patient serum	[106]

## 4. Research Progress of Induction of Immune Tolerance by Whey Protein

### 4.1. Whey Protein Hydrolysate Induces Immune Tolerance

Enzymatic hydrolysis is a widely used method to reduce the sensitization of allergen proteins because it can cleave peptide bonds in proteins and disrupt the epitope structure, leading to reduced antigenicity. However, differences in enzyme type, mode of hydrolysis, and extent of hydrolysis during hydrolysis can lead to variations in the peptide composition, residual antigenicity, and taste of the hydrolysate. A limited number of hypoallergenic infant formulas based on protein hydrolysates have been developed (Table 2). According to the MW of the hydrolysate, it can be divided into partially hydrolyzed formula (also known as moderately hydrolyzed formula, pHF) and extensively hydrolyzed formula (eHF) [115]. Shi Jiaqi et al. [116] used the CMA mouse model to evaluate pHF sensitization and found that it could effectively weaken whey protein sensitization and inhibit the occurrence of IgE-mediated immediate allergic reaction. Karen Knipping et al. [117] evaluated the sensitization potential of the newly developed extensive whey hydrolysate (eWH) using in vitro experiments and an in vivo CMA mouse model and showed that the newly developed eWH failed to induce whey-specific IgE antibodies in mice. Amina Chikhi et al. [118] used a Th2-biased mouse model mimicking high-risk patients to evaluate the ability of pHF-Ws to induce oral tolerance and showed that pretreatment with pHF-Ws only partially prevented CMA in Th2-biased mice and also showed that the use of a pHF obtained from whole whey was more effective in reducing CMA than whey hydrolysate. However, not all studies have shown that protein hydrolysates can establish IT. Tristan Bourdeau et al. [119] used the CMA Sprague Dawley Rats model to verify OIT of pWF and found that not all pHF-Ws can reduce sensitization and induce OIT.

### 4.2. Whey Protein Combined with Other Components Synergistically Induces Immune Tolerance

Studies on the synergistic induction of IT by whey protein combined with other components have also been reported. The anti-allergic effects of phenolic compounds have been associated with the inhibition of mast cell activation and degranulation [127,128], and also with the inhibition of FCεRI expression on the surface of mast cells [129]. Phenolic compounds can also alter protein sensitization by forming soluble and insoluble complexes with these macromolecules, thereby modifying epitopes or reducing bioavailability [130,131,132]. Whey protein isolate (WPI) complexed with caffeic acid (CA) or (−) epigallocatechin-3 gallate (EGCG) into WPI-CA and WPI-EGCG attenuated oral sensitization in C3H/HeJ mice through different mechanisms. The experimental results of Tassia B. Pessato et al. suggest that the complexation of whey proteins with CA and EGCG may be a promising strategy to induce oral tolerance [4]. Mengshan Liu et al. [120] carried out OIT experiments of polylactic acid-glycolic acid nanoparticles (PLGA-NPs) coated with screened β-lactoglobulin-derived peptide and TLR9 ligand CpG oligodeoxynucleotides. It was found that the oral administration of two β-LG peptides coated with PLGA-NPs was effective in inducing partial OIT to whey protein in a dose-dependent manner. On this basis, the team added TLR9 ligand CpG to continue to induce OIT in mice and found that β-LG -Pep+CpG/NP pretreatment altered DC phenotype, reduced Th2 development, and promoted Th1 and Treg microenvironments, which may contribute to the reduced development of allergen-specific CMA [121].

Studies on the induction of OIT by hypoallergenic infant formula supplemented with probiotics and prebiotics have also been reported. Raphaela Freidl et al. [122] studied allergenic activity and the ability to induce T cell and cytokine responses of IFs based on eHF supplemented with low galactose and Lactobacillus CECT5716 (LF). The results showed that this eHF could treat and prevent CMA by inducing specific T cell proliferation. Lindsey Otten et al. [123] analyzed the gastrointestinal tolerance of healthy full-term infants to IFs made of eHF or cow’s milk protein. The data show that eHF exhibits gastrointestinal tolerance as good as cow’s milk protein in healthy infants. Shujuan Jiang et al. [124] studied the immune effect of β-LG hydrolysis by L. cartarum AHQ-14 (H14) or L. bulgaricus BD0390 (H390) in a sensitized mouse model. It was found that H14 and H390 had a safe application value and therapeutic potential in sensitized animals and infants allergic to β-LG. Jean-Charles Picaud et al. [125] evaluated the safety and tolerability of two partially hydrolyzed whey protein (pHWP) containing a mixture of short-chain galactooligos (90%) and long-chain fructose oligos (10%) compared with cow’s milk protein formulas (IPFs). The results showed that pHWP was shown to support more adequate infant growth, equivalent compared with IPF, and was well immune tolerated and safe in healthy term infants. Chu Xiaojun et al. [126] using a BALB/c mouse sensitization model immuned with milk protein, studied the improvement effect of pHWP combined with different doses of Fructooligosaccharide (FOS) and Bifidobacterium animal Bb-12 (Bb-12) on CMA and the regulatory mechanism of IT. The results showed that pHWP combined with FOS and Bb-12 can effectively alleviate the symptoms of CMA in mice by IT, which may be related to blocking the secretion of IgE, CMP-sIgE, sIgG1, and histamine, inhibiting the proliferation of spleen cells, and upregulating TGF-β and INF-γ/IL-4. However, not all studies support the good results of extensively hydrolyzed whey formula in inducing IT. The results from a 3-year cohort study in the United Kingdom showed that extensively hydrolyzed casein formula containing L. rhamnosus Goldin was the most cost-effective, with a lower total cost, compared with amino acid-based formula, soy formula, and extensively hydrolyzed whey protein. Moreover, the proportion of children with no symptoms and IT 3 years after diagnosis is high [133].

## 5. Conclusions and Prospects

In summary, this article overviews the mechanism of OIT in the gastrointestinal tract and elaborates the molecular mechanism of Treg (Foxp3+CD4+T cells, Th3 regulates cells, and Tr1 cells), DCs, and intestinal flora on IT and the relationship among them. T cell epitopes of β-LG and α-LA in whey protein play an important role in inducing OIT. Research progress of the induction of immune tolerance by whey protein hydrolysate or whey protein combined with other components are overviewed and analyzed. However, current studies are mainly based on in vitro and in vivo experiments, and limited clinical studies have been reported. The clinical application of whey protein hydrolysate or hypoallergenic infant formula for the treatment of CMA is still in the exploratory stage. In the future, it is recommended that multicenter clinical trials be conducted to validate the OIT effect of whey protein hydrolysate or whey protein polyphenol (or probiotic) complex. Insufficient sample may limit the in-depth exploration of sub-group analysis, and larger studies are needed in the future to improve the reliability and accuracy of the results.

The standards of cohort studies on OIT are not completely consistent around the world, and the measures taken to manage CMA are also different. In addition, the update and statistics of epidemiological survey data are one of the directions of future research efforts. Partially hydrolyzed milk protein can be used as raw material for ordinary IFs, and extensively hydrolyzed milk protein can be used as raw material for formula food for special medical use. Relevant provisions are also found in the Codex standard for IF food (milk protein and its hydrolysate) [134], whereas the optimal conditions for the release of T cell epitopes from partially hydrolyzed milk protein or extensively hydrolyzed milk protein need to be studied in depth. In particular, the advantages of whey protein or its hydrolysates in inducing OIT are even more significant. The processes of immune induction and tolerance reinstatement in OIT are complicated. The novel immunological indicators need to be assessed as biomarkers in OIT. The ideal biomarkers are needed for easier and more efficient clinical assessment, which would be used to accurately predict treatment response, such as limited remission, transient state of desensitization, or sustained unresponsiveness, also, timely monitor therapeutic efficiency.

## Figures and Tables

**Table 2:** Summary of hypoallergenic or OIT hydrolyzed whey protein formulas.

Main Components	Research Methods	Results	Reference
pHWF	CMA BALB/c mouse model	Alleviate CMA	[116]
eWH	CMA C3H/HeOuJ mouse model	Establish OIT	[117]
pHWF	OIT BALB/c mouse model	Alleviate CMA	[118]
pHWF	CMA Sprague Dawley Rats model	OIT failed	[119]
WPI and phenolic compound—CA or EGCG	OIT C3H/HeOuJ mouse model	Attenuate oral sensitization	[4]
β-LG -derived peptides loaded PLGA nanoparticles	OIT C3H/HeOuJ mouse model	Establish OIT	[120]
β-LG -Pep and TLR9 ligand CpG loaded PLGA nanoparticles	OIT C3H/HeOuJ mouse model	Establish OIT	[121]
eHF, GOS, LF CECT 5716	Allergic infants T cell proliferation test	Reduce sensitization but retained T cell reactivity	[122]
eHF, GOS, LF CECT 5716	Infant clinical experiment	Establish OIT	[123]
β-LG hydrolysis by L. cartarum AHQ-14 or L. bulgaricus BD0390	OIT BALB/c mouse model	Establish OIT	[124]
pHWF, FOS, GOS	A Randomized, Double-Blind, Equivalence Trial	Establish OIT	[125]
pHWP+FOS+Bb-12	CMA BALB/c mouse model	Establish OIT	[126]

## Abbreviations

The following abbreviations are used in this manuscript:

CMA	Cow milk allergy
FA	food allergy
WAO	World Allergy Organization
DRACMA	Diagnostic and Action Criteria for cow’s milk allergy
α-LA	α-lactalalbumin
β-LG	β-lactoglobulin
OIT	oral immune tolerance
IT	immune tolerance
LP	lamina propria
GALT	gut-associated lymphoid tissue
MLN	mesenteric lymph nodes
PP	peyer’s patch
DCs	dendric cells
Treg	regulatory T cells
nTreg	natural regulatory T cells
iTreg	induced regulatory T cells
Foxp3	Forkhead box P3
Th2	T helper 2 cell
TGF-β	transforming growth factor-β
IL-10	interleukin-10
RA	retinoic acid
CMP-OIT	cow’s milk protein-specific induction of oral immune tolerance
APCs	antigen-presenting cells
TLR	Toll-like receptor
IFs	infant formula
PBMC	peripheral blood mononuclear cell
TCLs	T cell lines
MW	molecular weight
pHF	partially hydrolyzed formula powder
eHF	extensively hydrolyzed formula powder
eWH	extensive whey hydrolysate
PHF-Ws	partially hydrolyzed whey formula
WPI	whey protein isolate
CA	caffeic acid
EGCG	(−) epigallocatechin-3 gallate
pHWP	partially hydrolyzed whey protein
FOS	Fructooligosaccharide
Bb-12	Bifidobacterium animal Bb-12
